# Secreted Amyloid Precursor Protein-Alpha Promotes Arc Protein Synthesis in Hippocampal Neurons

**DOI:** 10.3389/fnmol.2019.00198

**Published:** 2019-08-14

**Authors:** Rhys W. Livingstone, Megan K. Elder, Maya C. Barrett, Courteney M. Westlake, Katie Peppercorn, Warren P. Tate, Wickliffe C. Abraham, Joanna M. Williams

**Affiliations:** ^1^Department of Anatomy, Brain Health Research Centre, Brain Research New Zealand, Rangahau Roro Aotearoa, University of Otago, Dunedin, New Zealand; ^2^Department of Biochemistry, Brain Health Research Centre, Brain Research New Zealand, Rangahau Roro Aotearoa, University of Otago, Dunedin, New Zealand; ^3^Department of Psychology, Brain Health Research Centre, Brain Research New Zealand, Rangahau Roro Aotearoa, University of Otago, Dunedin, New Zealand

**Keywords:** Arc/Arg3.1, sAPPα, plasticity, PKG, α7nACh, NMDA, Alzheimer’s disease, FUNCAT-PLA

## Abstract

Secreted amyloid precursor protein-α (sAPPα) is a neuroprotective and memory-enhancing molecule, however, the mechanisms through which sAPPα promotes these effects are not well understood. Recently, we have shown that sAPPα enhances cell-surface expression of glutamate receptors. Activity-related cytoskeletal-associated protein Arc (Arg3.1) is an immediate early gene capable of modulating long-term potentiation, long-term depression and homeostatic plasticity through regulation of α-amino-3-hydroxy-5-methyl-4-isoxazolepropionic acid receptor localization. Accordingly, we hypothesized that sAPPα may enhance synaptic plasticity, in part, by the *de novo* synthesis of Arc. Using primary cortical and hippocampal neuronal cultures we found that sAPPα (1 nM, 2 h) enhances levels of *Arc* mRNA and protein. Arc protein levels were increased in both the neuronal somata and dendrites in a Ca^2+^/calmodulin-dependent protein kinase II-dependent manner. Additionally, dendritic Arc expression was dependent upon activation of mitogen-activated protein kinase and protein kinase G. The enhancement of dendritic Arc protein was significantly reduced by antagonism of *N*-methyl-D-aspartate (NMDA) and nicotinic acetylcholine (α7nACh) receptors, and fully eliminated by dual application of these antagonists. This effect was further corroborated in area CA1 of acute hippocampal slices. These data suggest sAPPα-regulated plasticity within hippocampal neurons is mediated by cooperation of NMDA and α7nACh receptors to engage a cascade of signal transduction molecules to enhance the transcription and translation of Arc.

## Introduction

Secreted amyloid precursor protein-alpha (sAPPα) is a neuroprotective and neurotrophic protein, derived from the same parent protein as neurotoxic amyloid-ß. The levels of endogenous sAPPα are reduced in neurological disorders, including Alzheimer’s disease (AD; Lannfelt et al., 1995; Kim et al., 2009). By contrast, enhancement of sAPPα levels is protective against AD-associated memory impairments (Fol et al., 2016; Tan et al., 2018) and attenuates excitotoxic injury *in vivo* and *in vitro* (Mucke et al., 1996; Ryan et al., 2013). Further, sAPPα is able to facilitate long-term potentiation (LTP; Taylor et al., 2008; Moreno et al., 2015), stimulate neurite outgrowth (Clarris et al., 1994), and regulate spine morphology (Hick et al., 2015). Recently, it has been shown that the molecular mechanisms underpinning these actions include enhancement of glutamate receptor trafficking, synaptodendritic protein synthesis and new gene transcription (Claasen et al., 2009; Chasseigneaux et al., 2011; Ryan et al., 2013; Mockett et al., 2019), yet these and other mechanisms have not been fully explored.

Numerous studies have identified the importance of the immediate early gene (IEG) activity-regulated cytoskeletal-associated protein Arc (also referred to as activity-regulated gene 3.1, Arg3.1) in mediating synaptic changes associated with LTP, long-term depression (LTD) and homeostatic plasticity, which together permit the formation and maintenance of long term memories (Lyford et al., 1995; Guzowski et al., 2000; Plath et al., 2006; Messaoudi et al., 2007; Nakayama et al., 2016). Arc transcription is a well-established marker of plasticity (Grinevich et al., 2009; Izumi et al., 2011) and can be driven by activation of ionotropic, metabotropic, and enzyme-linked receptors (Kristensen et al., 2007; Bloomer et al., 2008; Waung et al., 2008; Peng et al., 2010; Gangarossa et al., 2011; Kumar et al., 2011; Kuipers et al., 2016; Chen et al., 2017). Interestingly, *Arc* mRNA is translated in both somata and dendrites of activated neurons (Steward and Worley, 2001; Steward et al., 2014). In dendrites, newly translated Arc protein associates with the *F*-actin-binding protein debrin A (Nair et al., 2017), and components of the clathrin-mediated endocytic machinery, dynamin-2 and endophilin-3 (Chowdhury et al., 2006). Indeed, Arc has been shown to promote internalization of GluA1- and GluA2-containing α-amino-3-hydroxy-5-methyl-4-isoxazolepropionic acid (AMPA) receptors (Chowdhury et al., 2006; Rial Verde et al., 2006), as well as Ca^2+^-permeable, homomeric GluA1-containing AMPARs; corroborated by a reduction in the rectification index of AMPA receptor-mediated miniature excitatory post-synaptic current amplitudes in Arc-overexpressing cortical neurons (DaSilva et al., 2016; Wall and Correa, 2018). Arc has also been shown to associate with CaMKIIβ, the so-called ‘inverse tag’ of inactive synapses, promoting AMPA receptor internalization (Okuno et al., 2012), as well as CaMKIIα (Husi et al., 2000) and stimulate neurite extension (Donai et al., 2003). While these data suggest a role for Arc in depotentiation or metaplasticity, new data indicate that Arc is released from neurons in virus-like caspids and thus may play a role in cell-to-cell communication (Pastuzyn et al., 2018).

Notably, while the receptor/s mediating the actions of sAPPα have not been conclusively identified, candidate receptors and downstream pathways overlap with those identified in relation to the regulation of Arc. Somatodendritic translation of *Arc* mRNA is dependent on Ca^2+^ signaling via ionotropic receptors, including the *N*-methyl-d-aspartate receptor (NMDA; Chen et al., 2017) and α7 nicotinic acetylcholine receptor (α7nAchR; Kristensen et al., 2007); these receptors are both candidates for mediating sAPPα’s actions (Taylor et al., 2008; Richter et al., 2018; Mockett et al., 2019). Furthermore, downstream signaling molecules such as protein kinase G (PKG), mitogen activated protein kinase (MAPK) and CaMKII have not only been shown to enhance *Arc* mRNA or regulate Arc protein expression (Huang et al., 2007; Gakhar-Koppole et al., 2008; Ota et al., 2010; Chasseigneaux et al., 2011), but also mediates the neuroprotective, neurotrophic and plasticity-enhancing effects of sAPPα (Furukawa et al., 1996a; Claasen et al., 2009; Mockett et al., 2019).

Based on the commonality in pathways regulated by sAPPα and those which enchance Arc expression, we hypothesized that heightening sAPPα levels would upregulate Arc expression. Using primary neuronal cultures, we found that exogenously delivered recombinant sAPPα (1 nM, 2 h) enhanced both Arc mRNA and protein through activation of both NMDA and α7nACh receptors, and that this effect is dependent on the activity of CaMKII, MAPK and PKG.

## Results

**FIGURE 1:**
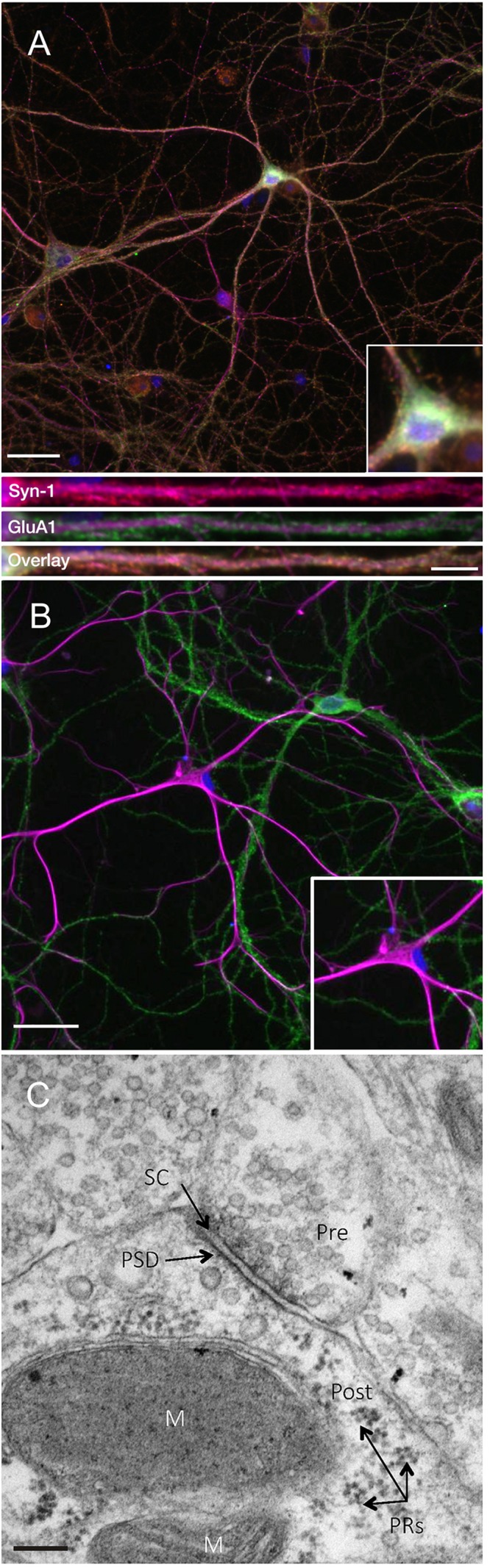
Primary cell cultures display normal expression of cellular and synaptic markers at DIV24-27. **(A)** Representative immunocytochemistry images of DIV 21-27 neurons show the colocalization of the presynaptic protein synapsin-1 (red) and the postsynaptic AMPA receptor subunit GluA1 (green) with MAP2-positive neurons (magenta) and nuclei (DAPI; blue) (scale bar = 50 μm). Lower panels show further magnified dendritic compartments (100 μm) from Synapsin-1 (top), GluA1 (middle) and the colocalization of both (bottom; scale bar = 10 μm). Primary cell cultures also show populations of **(B)** GFAP-positive astrocytes (magenta) closely associating with GluA1-positive neurons (green). Inset images show further magnified somatic compartments. **(C)** Representative electron micrograph showing the presence of synapses between neighboring primary hippocampal cells in culture. Pre- and postsynaptic regions were observed separated by a synaptic cleft. Pre, presynaptic terminal; Post, postsynaptic region; PRs, polyribosomes; PSD, postsynaptic density; SC, synaptic cleft; M, mitochondria. Scale bar = 100 nm.

In order to investigate the expression of the key plasticity protein Arc, we first sought to confirm that DIV24-27 primary neuronal cultures form mature synapses. Consistent with previous literature (Basarsky et al., 1994; Papa et al., 1995; Grabrucker et al., 2009), we found that our cultures coexpress the presynaptic marker synapsin-1 and the postsynaptic AMPA receptor subunit GluA1 on MAP2-positive neurons (Figure 1A). Co-expression was evident in both somatic and dendritic compartments, as previously observed (Richmond et al., 1996). Additionally, our cultures show populations of GFAP-positive astrocytes closely associated with GluA1-positive neurons (Figure 1B). This association has been shown to support the development of synapses *in vitro* (Jones et al., 2012). Further, ultrastructural analysis of our cultured neurons shows evidence of mature synapses (Figure 1C; Robert et al., 2012).

### sAPPα Facilitates an Increase in Arc mRNA Expression

**FIGURE 2:**
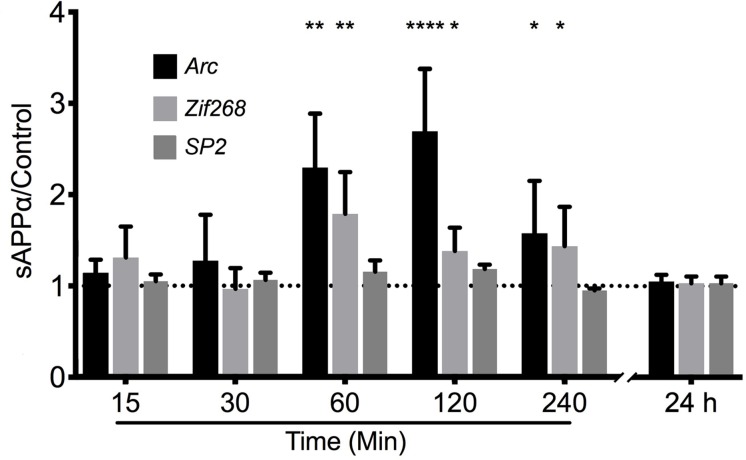
sAPPα promotes the transcription of *Arc* and *Zif268* mRNA. RT-qPCR showed that sAPPα (1 nM) promotes an increase in the expression of *Arc* mRNA in primary cortical cells in culture at 60, 120 min (*n* = 5) and 240 min (*n* = 4) relative to no-drug controls (*n* = 9; mean ± SEM). No significant change was detected at 15 or 30 min, nor 24 h (*n* = 4), nor was there a significant change in the negative control gene SP2 (*n* = 4). One sample *t*-tests; ^∗^*p* ≤ 0.05, ^∗∗^*p* ≤ 0.01, ^∗∗∗∗^*p* ≤ 0.0001.

To test the hypothesis that sAPPα may regulate Arc expression we investigated the ability of recombinant sAPPα (1 nM) to promote transcription of *Arc* mRNA in rat cortical neurons (DIV 24-27). As a positive control we also assessed the levels of the known sAPPα-responsive IEG *Zif268* (Chasseigneaux et al., 2011; Penke et al., 2011; Ryan et al., 2013). As a negative control we assessed the levels of the constitutively expressed transcription factor SP2 (Suske, 1999). We found that treatment with sAPPα facilitated a slowly developing increase in the levels of *Arc* mRNA (Figure 2). There was no significant fold change in either *Arc* or *Zif268* mRNA after 15 or 30 min of sAPPα incubation (*Arc*: 15 min: 1.27 ± 1.00, *p* = 0.35; 30 min: 1.14 ± 0.28, *p* = 0.49; *Zif268*: 15 min: 0.96 ± 0.45, *p* = 0.28; 30 min: 1.30 ± 0.68, *p* = 0.30), but following 60 min exposure the levels of both *Arc* (2.29 ± 1.32, *p* = 0.01) and *Zif268* (1.78 ± 1.02, *p* = 0.01) mRNA increased significantly relative to no-drug controls. Interestingly, while both *Arc* (2.69 ± 1.53, *p* < 0.0001) and *zif268* mRNA remained significantly elevated at 120 min (1.38 ± 0.57, *p* = 0.04), and 240 min (*Arc*: 1.575 ± 1.15, *p* = 0.03; *Zif268*: 1.434 ± 0.86, *p* = 0.03), both Arc and Zif268 mRNA expression appeared to decline at this timepoint and was not significantly different from controls 24 h later (Arc: 1.01 ± 0.14, *p* = 0.42; Zif268: 1.03 ± 0.14, *p* = 0.15). At no point was SP2 significantly different from controls (15 min: 1.04 ± 0.15, *p* = 0.20; 30 min: 1.14 ± 0.04, *p* = 0.27; 60 min: 1.15 ± 0.24, *p* = 0.16; 120 min: 1.18 ± 0.09, *p* = 0.07; 240 min: 0.94 ± 0.04, *p* = 0.29; 24 h: 1.03 ± 0.14, *p* = 0.54).

### sAPPα Mediated New Synthesis of Arc Protein: Detected Using FUNCAT-PLA

**FIGURE 3:**
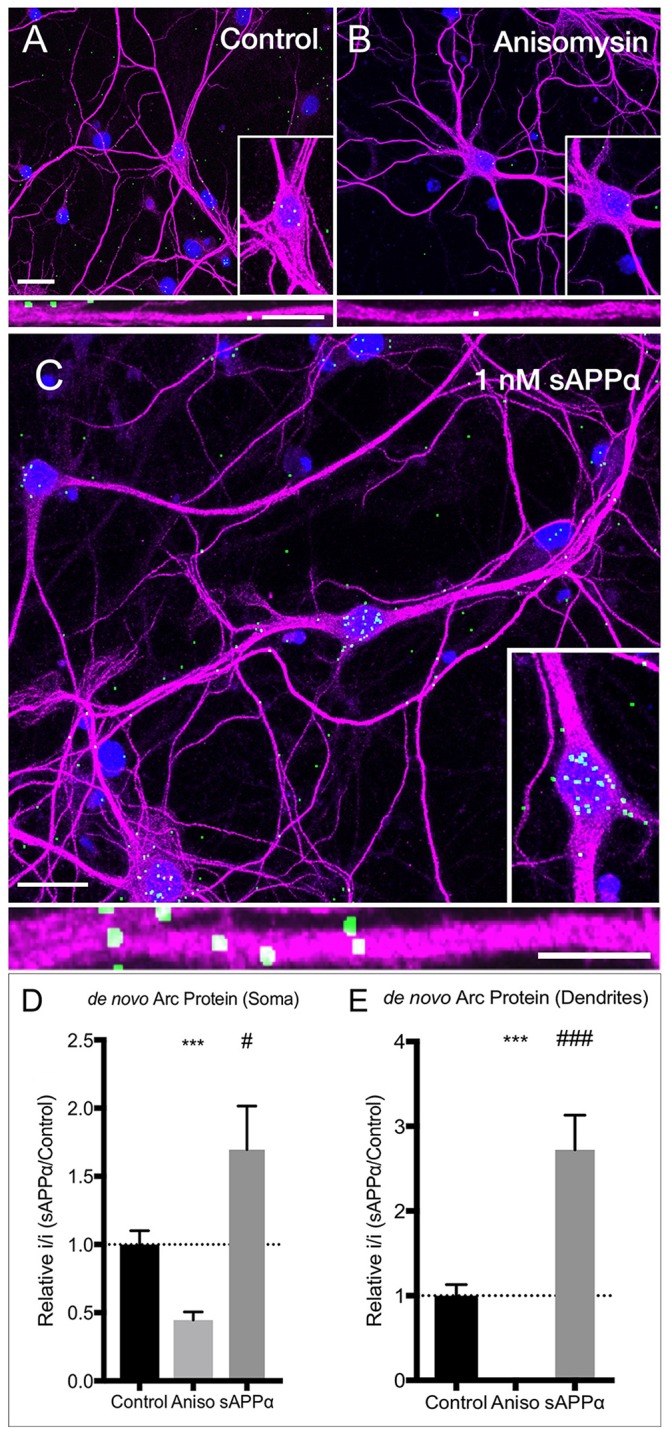
sAPPα increases somatic and dendritic expression of *de novo* Arc protein. Representative images show neurons (MAP2 positive neurons; magenta) expressing FUNCAT-PLA signal (green puncta) representing newly synthesized Arc protein in **(A)** no-drug control cells, **(B)** anisomycin-treated, and **(C)** sAPPα-treated (1 nM, 2 h) primary hippocampal cultures. Nuclei are stained blue (DAPI). The relative integrated intensity of **(D)** somatic and **(E)** dendritic signal from treatment groups is expressed as drug/average of control and presented as mean ± SEM (*n* = 25–34 cells from three independent experiments). Co-incubation of AHA with anisomycin inhibited Arc protein synthesis. Incubation with sAPPα significantly increased the expression of *de novo* synthesized Arc protein in the somata and dendrites. Images show whole cell (scale bar = 50 μm), and magnified somatic (inset, bottom right) and dendritic (50 μm; lower panels; scale bar = 10 μm). Outliers were removed from each experiment prior to amalgamation using Grubb’s tests, and normality was detected by D’Agostino and Pearson omnibus normality tests. Significance was calculated on data expressed relative to control by use of one sample *t*-tests; hashes (#) indicate significance between control and sAPPα-treated, asterisks (^∗^) indicate significance between control and anisomycin treated, ^#^*p* = 0.038, ^∗∗∗/###^ *p* ≤ 0.0001.

Next, we directly visualized *de novo* Arc protein synthesis in response to sAPPα using fluorescence non-canonical amino acid tagging with proximity ligation assays (FUNCAT-PLA: Figure 3). Here, we found that sAPPα (1 nM, 2 h) induced a highly significant increase in newly synthesized Arc protein in both the dendrites (2.72 ± 0.41, *p* ≤ 0.0001; Figures 3C–E) and somata (1.69 ± 0.32, *p* = 0.03) of cultured hippocampal neurons. Co-incubation with protein synthesis inhibitor anisomycin eliminated labeling (somata, 0.44 ± 0.06, *p* ≤ 0.0001; dendrites, 0.004 ± 0.004, *p* ≤ 0.0001; Figures 3B,D,E), confirming the punctate signal was specific for *de novo* synthesized protein. Interestingly, a few puncta were found located outside the MAP2 positive cells. These may reflect a low level of background stain or the synthesis of Arc within astorcytes (Rodriguez et al., 2008).

### sAPPα Facilitates Arc Protein Expression in Primary Hippocampal Neurons

**FIGURE 4:**
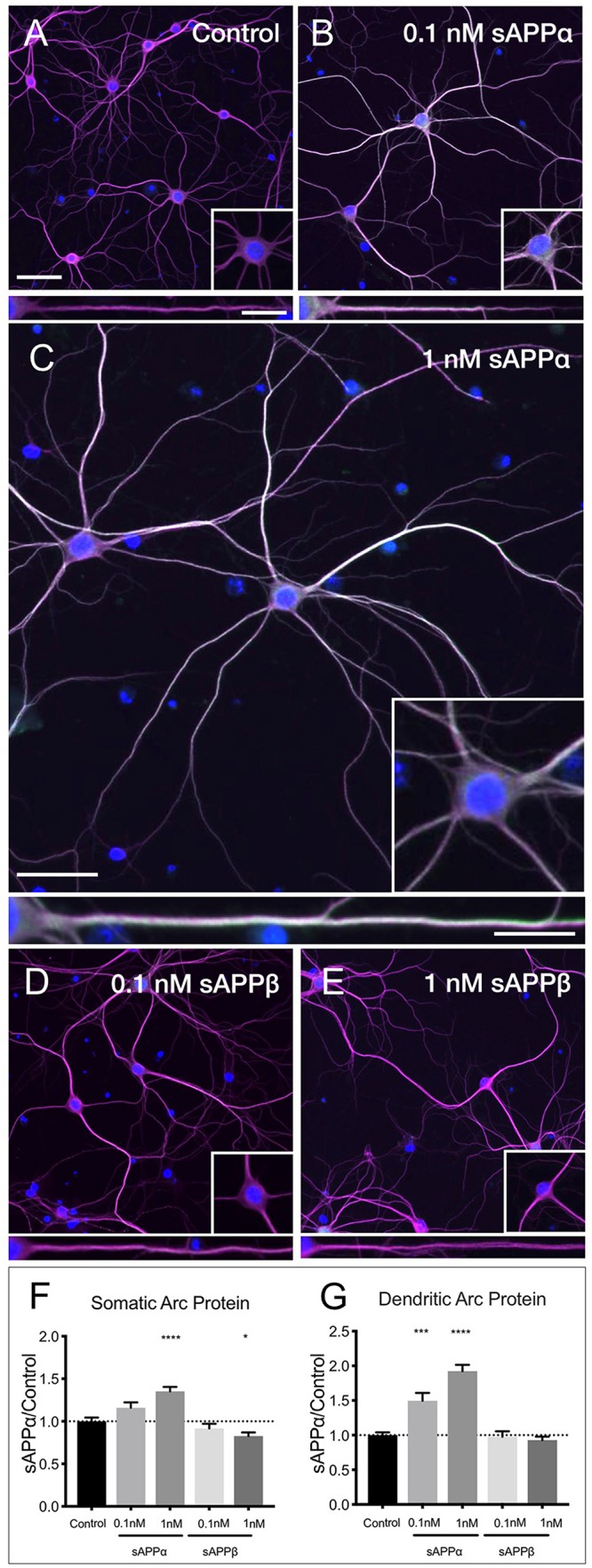
sAPPα promotes Arc protein expression in a concentration- dependent manner. Representative images showing Arc protein levels in **(A)** no drug control, **(B)** 0.1 nM sAPPα, **(C)** 1 nM sAPPα, **(D)** 0.1 nM sAPPβ and **(E)** 1 nM sAPPβ-treated primary hippocampal neurons. **(F)** Average data showing 1 nM sAPPα promotes an increase, and 1 nM sAPPβ a modest decrease in Arc protein expression in the somata. **(G)** Average data showing 0.1 and 1 nM sAPPα promotes an increase in dendritic Arc protein. Data are expressed as mean ± SEM from ≥4 experiments. 0.1 nM: *n* = 40 cells; 1 nM: *n* = 80 cells. Significance was calculated using a Kruskal–Wallis one-way ANOVA with Dunn’s multiple comparisons test. ^∗^*p* = 0.0378, ^∗∗∗^*p* = 0.0002, ^∗∗∗∗^*p* ≤ 0.0001. Representative images show neuronal somata and dendritic projections (MAP2; magenta), Arc protein (green), nuclei (DAPI; blue), (scale bar = 50 μm), and magnified somatic (inset, bottom right) and dendritic (100 μm; lower panels; scale bar = 10 μm) compartments.

Using western blot analysis we showed that the anti-Arc primary antibody detected a signal band of the expected molecular weight (Supplementary Figure 1). Next, using this antibody in immunocytochemistry, we showed that application of sAPPα significantly increased Arc protein expression in a concentration dependent manner. Specifically, 0.1 nM sAPPα was sufficient to significantly increase dendritic Arc expression (1.35 ± 0.05, *p* = 0.0002), although it did not affect somatic Arc protein expression (1.15 ± 0.39, *p* = 0.24; Figures 4A,B,F,G). Conversely, 1 nM sAPPα significantly increased both somatic (1.35 ± 0.46, *p* ≤ 0.0001) and dendritic (1.92 ± 0.81, *p* = 0.0003) Arc protein expression relative to controls (Figures 4A,C,F,G; see Supplementary Figure 2 for Arc-only gray scale image). Further, using confocal microscopy we were able to show Arc expression to be punctate in dendrites, typical of synapse-associated proteins (Supplementary Figure 3).

sAPPβ differs from sAPPα by 16 C-terminal amino acids. sAPPα is derived following cleavage of amyloid precursor protein by a-secretase activity, whereas sAPPβ is liberated by β-secretase 1. sAPPβ has been described as 100-fold less effective in ameliorating excitotoxicity and attenuating glucose deprivation compared to sAPPα (Furukawa et al., 1996b; Turner et al., 2007), but appears to remain unaltered in AD (Sennvik et al., 2000). Accordingly, we next examined the effect of sAPPβ on Arc expression. We found that sAPPβ did not affect Arc protein in dendrites (0.1 nM: 0.97 ± 0.49; 1 nM: 0.92 ± 0.45, *p* ≥ 0.99, respectively; Figures 4D–G). Interestingly, 0.1 nM sAPPβ did not affect Arc protein expression in the somata (0.91 ± 0.34, *p* ≥ 0.99), while 1 nM sAPPβ resulted in a small but significant decrease in somatic Arc expression (0.82 ± 0.38, *p* = 0.03).

**FIGURE 5:**
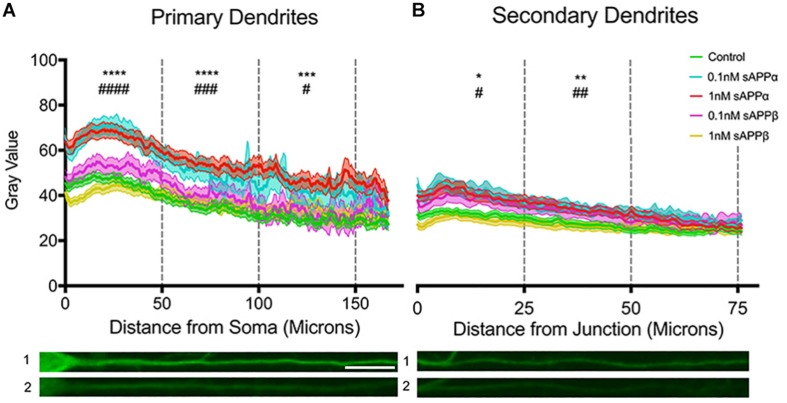
Arc protein expression increases throughout primary and secondary dendrites. Average fluorescent intensity of Arc expression throughout **(A)** primary and **(B)** secondary dendrites (mean ± SEM, *n* = 40–141). Significance was determined by averaging dendritic gray value within each cell and is expressed per treatment group within **(A)** 50 and **(B)** 25 microns. Asterisks (^∗^) and hashes (#) indicate significance found between 1 and 0.1 nM sAPPα, respectively, relative to controls. Representative fluorescence images illustrate Arc protein expression (green) within primary (A1,2) and secondary (B1,2) dendrites in the presence of 1 nM sAPPα (A1,B1) or no drug (A2,B2). Significance was determined by Kruskal–Wallis One-Way ANOVA and Dunn’s multiple comparisons test. ^∗/#^*p* = ≤ 0.05 ^∗∗/##^*p* = ≤ 0.005, ^∗∗∗/###^*p* = 0.0005, ^∗∗∗∗/####^*p* = ≤ 0.0001. Scale bar = 50 μm.

To extend our findings that sAPPα (1 nM, 2 h) increased dendritic Arc expression, we assessed fluorescence intensity levels and distribution of Arc protein throughout the dendrites according to the method of Gumy et al. (2017). Using this approach we found that dendritic Arc expression was significantly increased throughout primary dendrites in the initial 50 μm (control: 40.06 ± 14.93; 0.1 nM: 63.82 ± 20.1, *p* ≤ 0.0001; 1 nM: 62.04 ± 22.6, *p* ≤ 0.0001), middle 50 μm (control: 32.43 ± 15.69; 0.1 nM: 52.91 ± 25.57 *p* = 0.0005; 1 nM: 52.89 ± 22.29, *p* ≤ 0.0001) and final 50 μm segment of primary dendrites (control: 27.09 ± 11.58; 0.1 nM; 43.72 ± 22.3, *p* ≤ 0.0001; 1 nM 48.09 ± 24.17, *p* = 0.0311; Figure 5A). Increased Arc expression in secondary dendrites was observed in the initial 25 μm (control: 32.54 ± 13.55; 0.1 nM: 45.22 ± 19.61, *p* = 0.01; 1 nM: 43.73 ± 21.08, *p* = 0.01) and middle 25 μm dendritic segment (control: 27.03 ± 14.08; 0.1 nM: 37.64 ± 15.19, *p* = 0.002; 1 nM: 36.63 ± 17.49, *p* = 0.004; Figure 5B), however, Arc expression was not significantly altered in the final 25 μm of secondary dendrites (control: 24.85 ± 12.25; 0.1 nM: 31.99 ± 13.06, *p* = 0.26; 1 nM: 30.06 ± 14.26, *p* = 0.06).

**FIGURE 6:**
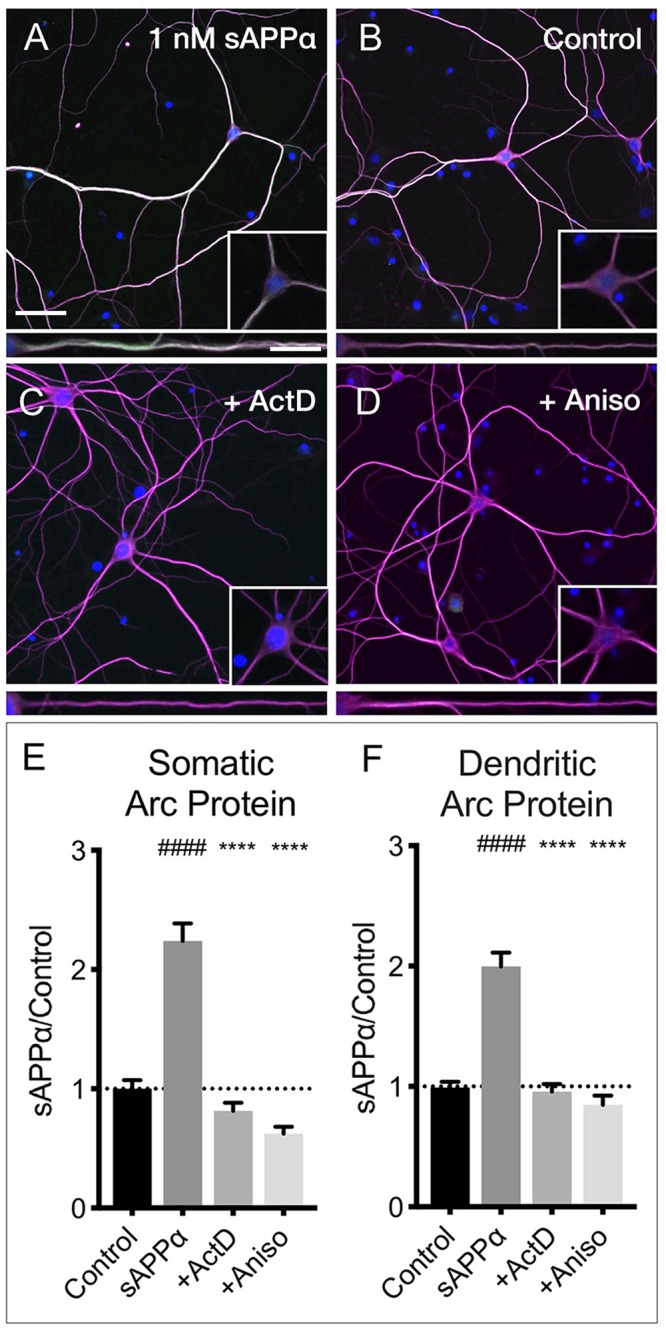
Arc protein expression is prevented by inhibitors of transcription and translation. Representative images showing Arc protein levels in **(A)** sAPPα (1 nM, 2 h, *n* = 157) and **(B)** no drug control (*n* = 115 cells) conditions. sAPPα-mediated Arc increase is inhibited with co-application of **(C)** Actinomycin-D (*n* = 104) or **(D)** Anisomycin (*n* = 30) in the **(E)** somata and **(F)** dendrites. Significance was calculated using a Kruskal–Wallis one-way ANOVA with Dunn’s multiple comparisons test. ^∗∗∗∗/####^*p* ≤ 0.0001. Representative images show neurons (magenta), Arc protein (green), nuclei (DAPI; blue) (scale bar = 50 μm) and magnified somatic (inset, bottom right) and dendritic (100 μm; lower panels; scale bar = 10 μm).

The sAPPα-induced increase in Arc protein expression in both somatic and dendritic compartments (Figures 6A,B,E,F) was blocked by co-application of the transcription inhibitor actinomycin-D (10 μM; somata: 0.81 ± 0.68; dendrites: 0.95 ± 0.61, respectively; *p* < 0.0001; Figures 6C,E,F). Additionally, co-application of the translation inhibitor anisomycin (40 μM) also eliminated the effect (somata: 0.62 ± 0.30; dendrites: 0.84 ± 0.41, *p* < 0.0001; Figures 6D–F). Together, these findings show that sAPPα promotes both Arc transcription and translation.

### sAPPα-Induced Arc Protein Expression Is Dependent on CaMKII, MAPK, and PKG Signaling

**FIGURE 7:**
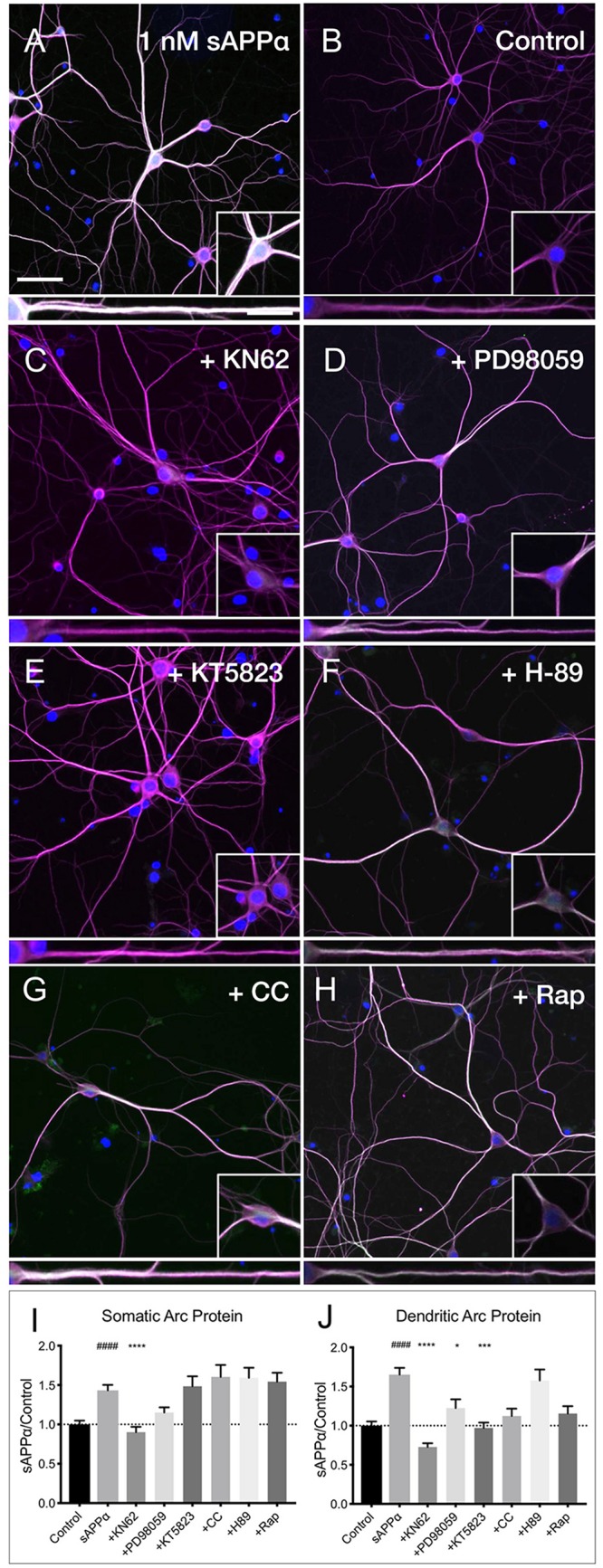
Arc protein expression in response to sAPPα is affected by kinase inhibitors. Representative images showing **(A)** sAPPα (1 nM, 2 h, *n* = 125) promotes an increase in the expression of Arc protein in cultured neurons relative to **(B)** no drug controls (*n* = 134). Cells were simultaneously treated with **(C)** KN62 (*n* = 51), **(D)** PD98059 (*n* = 51), and **(E)** KT5823 (*n* = 42), **(F)** H-89 dihydrochloride (*n* = 40), **(G)** Chelerythrine chloride (*n* = 30), or **(H)** Rapamycin (*n* = 40) and Arc levels in the **(I)** somata and **(J)** dendrites was measured. Data are expressed as mean ± SEM from ≥3 experiments. Significance was calculated using a Kruskal–Wallis one-way ANOVA with Dunn’s multiple comparisons test. Hashes (#) indicate significance between control and sAPPα-treated; asterisks (^∗^) indicate significance between sAPPα- and inhibitor-treated; ^####^*p* ≤ 0.0001, ^∗^*p* = 0.0428, ^∗∗∗^*p* = 0.0001, ^∗∗∗∗^*p* ≤ 0.0001. Representative images show neurons (magenta), Arc protein (green), DAPI (blue) (scale bar = 50 m), and magnified somatic (inset, bottom right) and dendritic (100 μm; lower panels; scale bar = 10 μm) compartments.

Previous research has identified CaMKII, MAPK, and PKG as key downstream kinases regulating sAPPα-mediated protein synthesis in synaptoneurosomes (Claasen et al., 2009). Here we sought to determine whether the sAPPα-induced expression of Arc protein utilizes these same signaling kinases. We observed that the sAPPα-mediated increase in Arc protein in the somata (Figures 7A,B,I,J) was suppressed following inhibition of CaMKII by KN62 (10 μM; 0.90 ± 0.48, *p* ≤ 0.0001; Figures 7C,I,J). Somatic Arc protein expression was also attenuated by the MAPK inhibitor PD98059 (50 μM; 1.15 ± 0.46, *p* = 0.47; Figures 7D,I,J), but not by the PKG inhibitor KT5823 (10 μM; 1.48 ± 0.82, *p* ≥ 0.99; Figures 7E,I,J), the PKC inhibitor chelerythrine chloride (1 μM; 1.60 ± 0.83, *p* ≥ 0.99; Figures 7F,I,J), the PKA inhibitor H-89 dihydrochloride (10 μM; 1.59 ± 0.80 *p* ≥ 0.99; Figures 7G,I,J), or the mTOR inhibitor rapamycin (20 nM; 1.54 ± 0.71, *p* ≥ 0.99; Figures 7H,I,J). Interestingly, by contrast dendritic Arc protein expression was significantly reduced via inhibition of CaMKII (0.72 ± 0.34, *p* ≤ 0.0001; Figures 7C,I,J), MAPK (1.22 ± 0.80, *p* = 0.04; Figures 7D,I,J), and PKG (0.97 ± 0.45, *p* ≥ 0.0001; Figures 7E,I,J), but it remained unaffected by inhibition of PKC (1.12 ± 0.51, *p* = 0.08; Figure 7F), PKA (1.57 ± 0.88, *p* ≥ 0.99; Figures 7G,I,J), and mTOR (1.15 ± 0.58, *p* = 0.051; Figures 7H,I,J). These results suggest that activation of CaMKII, MAPK, and PKG is necessary to facilitate sAPPα-induced Arc expression, but that different regulatory mechanisms may exist in somatic and dendritic compartments in primary hippocampal neurons.

### sAPPα-Induced Dendritic Arc Protein Expression Is Dependent on Activation of NMDA- and α7nACh Receptors

**FIGURE 8:**
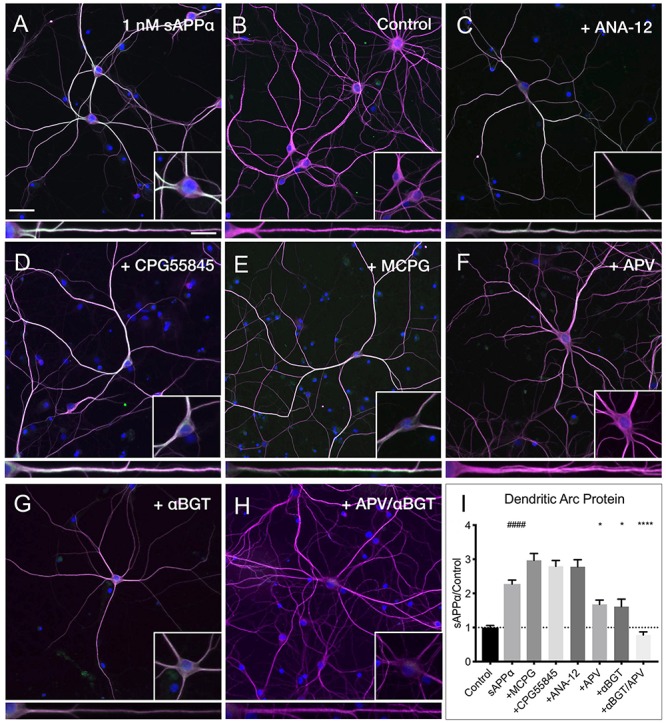
Arc protein expression is dependent on NMDA and α7nAch receptors. Representative images showing **(A)** sAPPα (1 nM, 2 h, *n* = 80) promotes and increase in the expression of Arc protein in cultured hippocampal neurons relative to **(B)** no drug controls (*n* = 80). Co-incubation of sAPPα with **(C)** ANA-12 (*n* = 40), **(D)** CPG55845 (*n* = 40), and **(E)** MCPG (*n* = 40) had no effect on sAPPα-induced Arc expression. Co-incubation with **(F)** APV (*n* = 40), or **(G)** αBGT (*n* = 30) significantly reduced Arc protein expression, while co-incubation with both **(H)** APV and αBGT (*n* = 30) fully eliminated this effect in the **(I)** dendrites. Outliers were removed from each experiment prior to amalgamation using Grubb’s tests, and normality was detected by D’Agostino and Pearson omnibus normality tests. Data are expressed as mean ± SEM from ≥3 experiments. Significance was calculated using a Kruskal–Wallis one-way ANOVA with Dunn’s multiple comparisons test. Hashes (#) indicate significance between control and sAPPα-treated; asterisks (^∗^) indicate significance between sAPPα- and antagonist-treated; ^####^*p* ≤ 0.0001, ^∗^*p* = 0.0163, ^∗∗∗∗^*p* ≤ 0.0001. Representative images show neurons (magenta), Arc protein (green), DAPI (blue) (scale bar = 50 μm), and magnified somatic (inset, bottom right) and dendritic (100 μm; lower panels; scale bar = 10 μm) compartments.

Despite many studies, the cell surface receptor(s) which transduce the sAPPα signal are yet to be conclusively identified, though specific candidates have emerged (Rice et al., 2017, 2019; Richter et al., 2018; Mockett et al., 2019). Here, we pharmacologically inhibited likely candidates mediating sAPPα’s plasticity-promoting effects and observed the effect on sAPPα-induced Arc protein levels (Figure 8). We found that application of antagonists targeting GABA_B_ (CPG55845; 50 μM), TrkB (ANA-12; 100 μM), or mGluRI/II receptors (MCPG; 500 μM) had no significant effect on dendritic Arc protein expression (Figures 8A,B,I) following sAPPα treatment (1 nM, 2 h; CPG55845: 2.79 ± 1.08, *p* ≥ 0.99, Figures 8C,I; ANA-12: 2.78 ± 1.29, *p* ≥ 0.99, Figures 8D,I; MCPG: 2.29 ± 1.27, *p* ≥ 0.99, Figures 8E,I). However, Arc protein expression was significantly reduced following antagonism of NMDA receptors by AP5 (50 μM; 1.67 ± 0.78, *p* = 0.01; Figures 8F,I) and α7nAch receptors with α-bungarotoxin (10 nM; 1.61 ± 1.18, *p* = 0.0006; Figures 8G,I). Combined antagonism of NMDA and α7nAch receptors completely abolished sAPPα-mediated Arc expression (0.78 ± 0.49, *p* ≤ 0.0001; Figures 8E,I). These results suggest a novel mechanism whereby synergistic action between NMDA and α7nAch receptors governs an enhancement in sAPPα-mediated Arc protein expression.

### sAPPα Increases CREB Phosphorylation and Arc Protein Levels in Area CA1 of Acute Hippocampal Slices

**FIGURE 9:**
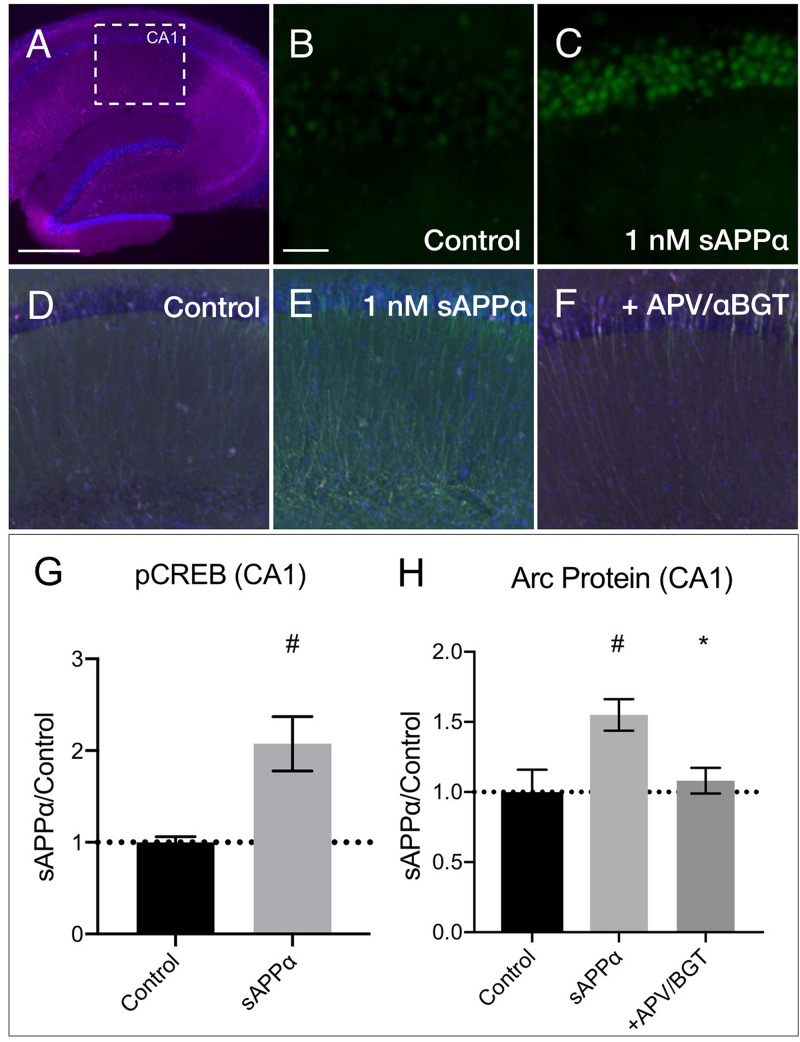
sAPPα increases in CREB phosphorylation and Arc protein in acute hippocampal slices. **(A)** A representative transverse section of an acute hippocampal slice, with a subregion of area CA1, used for quantitative analysis, outlined by a white dotted box (neurons: MAP2/magenta; nuclei: DAPI/blue; imaged at 4x magnification; scale bar = (500 μm). Relative to **(B)** no drug controls, **(C)** incubation of slices with sAPPα (1 nM, 15 min, *n* = 2 rats, 4 slices) increased pCREB_ser133_ (green; imaged at 20x magnification; scale bar = 100 μm) in the PCL of CA1. Relative to **(D)** no drug controls, **(E)** incubation of slices with sAPPα (1 nM, 2 h, *n* = 3 rats, 4 slices) significantly increased Arc protein expression (green; imaged at 4x magnification) in area CA1. Co-incubation of sAPPα with **(F)** APV and αBGT (*n* = 3 rats, 4 slices) attenuated this effect. Normality was detected by Shapiro–Wilk normality tests. Data are expressed as mean ± SEM. Significance of pCREB **(G)** and **(H)** Arc protein expression was calculated using a students *t*-test, and one-way ANOVA with Šidák’s multiple comparisons test, respectively. Hashes (#) indicate significance between control and sAPPα-treated; asterisks (^∗^) indicate significance between sAPPα- and antagonist-treated; ^#^*p* ≤ 0.05, ^∗^*p* = 0.04. CA1, cornu ammonis 1; PCL, pyramidal cell layer.

We next examined whether sAPPα could modulate transcription and Arc expression in acute hippocampal slices. Focusing on area CA1 (Figure 9A), we found that sAPPα (1 nM, 15 min) significantly increased phosphorylated cAMP element binding protein at serine 133 (pCREB) levels, a marker of transcriptional regulation as well as NMDAR signaling (Xia et al., 1996) (2.08 ± 0.60, *p* = 0.01; Figures 9B,C,G). We also showed that sAPPα (1 nM, 2 h) significantly increased Arc expression (1.55 ± 0.22, *p* = 0.02; Figures 9D,E,H) and that this effect was attenuated by co-incubation with NMDA and α7nAch antagonists APV and αBGT (1.08 ± 0.18, *p* = 0.042; Figures 9F,H).

## Materials and Methods

### Animals

Sprague-Dawley rat pups (male or female, P0-P1) were sourced from a breeding colony maintained at the Hercus Taieri Resource Unit by the University of Otago (Dunedin, New Zealand), or at the Max Planck Institute for Brain Research (Frankfurt, Germany). All experimental protocols conducted in New Zealand were approved by the University of Otago Animals Ethics Committee and conducted in accordance with New Zealand Animal Welfare Legislation under the ethics approval ET18/15 and AUP-18-136 for cell culture work and DET19/16 for all acute slice work. All experiments conducted in Germany were compliant with German animal care guidelines, and Max Planck Society guidelines, and were approved by local authorities. The preparation of primary hippocampal cultures followed a modified protocol based on Banker and Goslin (1998) and Kaech and Banker (2006). Cortex and hippocampi from Sprague-Dawley rat pups were dissociated using papain (Sigma) and plated at a low density on glass-bottomed culture dishes (40,000 cells/cm^2^; Mattek) for immunolabelling, or at 300,000 cells/well of a 6-well plate for lysis and subsequent protein or RNA extraction and RT-qPCR. Cells were cultured in Neurobasal A medium (Life Technologies #10888-022), supplemented with B27 (Life Technologies, #17504-001) and Glutamax (Life Technologies, #35050-061) at 37°C/5% CO_2_ for 24–27 days *in vitro* (DIV). Control samples were collected at the same time as treated cells in matched sets of culture dishes (immunocytochemistry, FUNCAT-PLA) or wells (RT-qPCR; western blot). All experiments conducted on acute tissue were prepared from young adult male Sprague-Dawley rats (42–56 days), as described previously (Mockett et al., 2019).

### Drugs and Reagents

#### Inhibitors

KN62 (Tocris, #1277), PD98059 (Tocris, #1213), KT5823 (Tocris, #1289), Chelerythrine chloride (Tocris, #1330), H-89 dihydrochloride (Calbiochem, #371963), Rapamycin (Tocris, # 1292); Actinomycin-D (Tocris, #1229), Anisomycin (Sigma, #A9789) Sodium Fluoride (NaF; Sigma #S7920), Phenylmethylsulfonyl Fluoride (PMSF; Sigma, #P-7626) Okadaic Acid (Tocris, #1136).

#### Antagonists

CGP 55845 hydrochloride (Tocris, #1248), ±α-Methyl-4-carboxyphenylglycine (Sigma, #M-4796), ANA-12 (Tocris, #4781), D(−)-2-Amino-5-phosphonopentanoic acid (APV; Sigma, #A8054), α-Bungarotoxin (αBGT; Abcam, # ab120542).

sAPPα and sAPPβ production and purification was carried out according to the protocols developed by the Tate lab (Turner et al., 2007).

### Immunocytochemistry

For experiments examining the effect of treatments, DIV24-27 primary hippocampal neurons were treated with sAPPα (0.1–1 nM, 2 h), sAPPβ (0.1–1 nM, 2 h) or culture media only. Following incubation, cells were fixed in 4% paraformaldehyde in PBS (pH 7.4; 20 min) supplemented with 1 mM MgCl_2_ and 0.1 mM CaCl_2_ (PBS-MC), and permeabilized with 0.5% Triton X-100 in PBS (pH 7.4; 15 min). Cells were then blocked in 4% normal goat serum in PBS (pH 7.4) for 1 h at room temperature (RT). Cells were incubated with primary antibodies of interest (2 h, RT), followed by 3 × 5 min washes (PBS, pH 7.4) and incubation in appropriate secondary antibody (30 min, RT), followed by 3 × 5 min washes (PBS, pH 7.4).

### Primary and Secondary Antibodies

#### Primary Antibodies

Rabbit anti-Arc polyclonal (1:1000, synaptic systems, #156003), mouse anti-Biotin monoclonal (1:1000, Sigma, #B7653), guinea-pig anti-MAP2 polyclonal (1:1000, synaptic systems, #188004; monoclonal, 1:1000, Abcam, #AB11267), rabbit anti-pCREB (Ser133) monoclonal (1:500, cell signaling, #9198), mouse anti-Synapsin-1 monoclonal (1:1000, synaptic systems, #106011), rabbit anti-GluA1 polyclonal (1:1000, Abcam, #AB31232), mouse anti-GFAP monoclonal (1:1000, Abcam, #AB10062), mouse anti-αTubulin monoclonal (1:4000; Abcam, #AB7291).

#### Secondary Antibodies and Fluorescent Stains

Goat anti-Guinea Pig Alexa Fluor 488 (1:1000, Thermofisher, #A11073), Goat anti-rabbit Alexa Fluor 555 (1:1000, Invitrogen, #A21429), Goat anti-mouse Alexa Fluor 647 (1:1000, Invitrogen, #A21236), Donkey anti-mouse PLAminus probe (1:10, Sigma-Aldrich, #DUO92004), Donkey anti-rabbit PLAplus probe (1:10, Sigma-Aldrich, #DUO92002), DAPI (1:1000, Thermofisher, #D1306), Duolink detection reagent Texas Red (1:5, Sigma-Aldrich, #DUO92008), DAPI (1:1000, Thermofisher, #D1306), goat anti-mouse IRDye 800CW (1:10,000, Licor, #926-32210), goat anti-rabbit IRDye 680RD (1:10,000, Licor, #926-68071).

### RT-qPCR

Total RNA was extracted from cortical cell cultures prepared in 6-well plates at a density of 31,500 cells/cm2. Tissue was lysed by using 350 μl Buffer RL (Norgen Biotek Corp., #17200), RNA was bound to spin columns (Norgen Biotek Corp., #17200) by centrifugation, followed by an on-column DNA removal. Purified RNA was eluted and quantified by spectrometry (Nanodrop ND-1000 v3.8.1 and associated software (Thermofisher Scientific).

Primers:

Zif268: Fwd-GGGAGCCGAGGGAACAA, Rev-CGTTATTC AGAGCGATGTCAGAA; Arc: Fwd-AGCAGAATCAGAGATG GCCG, Rev-TGAATCACTGCTGGGGGC;

HPRT: Fwd-TGACACTGGTAAAACAATGCA, Rev-GGGA GCCGAGCGAACAA;

SP2: Fwd-CAGCCTGGGGAGAAACGGCG, Rev-GCCCTG CTCCCCAGACCTCTT. cDNA libraries were created from total RNA using SuperScript III RT (Invitrogen). RT-qPCR was performed using SYBR Green Master Mix (Thermo Fisher Scientific).

### FUNCAT-PLA

FUNCAT-PLA labeling of newly synthesized proteins was conducted according to a previously published protocol (Dieterich et al., 2007; tom Dieck et al., 2015). Cells were incubated in 4 mM L-azidohomoalanine (AHA, courtesy of the Schuman laboratory, Max Planck Institute for Brain Research, Frankfurt) in the presence or absence of sAPPα or anisomycin. Following incubation, cells were washed with PBS-MC (pH 7.4), fixed in PFA in PBS-MC (pH 7.4), and permeabilized with 0.5% Triton X-100 in PBS (pH 7.4). Azide-labeled newly synthesized proteins were alkylated with biotin-linked alkyne via a copper-mediated click reaction. Click reaction mixture comprised of 200 μM triazole ligand [Tris ((1-benzyl-1H-1,2,3-triazol-4-yl)methyl) amine; TBTA, Aldrich], 500 μM TCEP (Tris(2-carboxyethyl)phosphine hydro-chloride, Thermo Scientific), 25 μM Biotin-PEG4-alkyne (Biotin alkyne, Aldrich) and 200 μM CuSO4 in PBS pH 7.8 was incubated on cells overnight at RT. For detection of *de novo* Arc protein, cells were incubated with anti-biotin, anti-Arc antibodies diluted in 4% normal goat serum. Donkey anti-mouse PLA^minus^, and donkey anti-rabbit PLA^plus^ probes were applied, followed by ligation and amplification with Duolink detection reagent Texas Red according to the manufacturer’s instructions. Neuronal somata, dendrites and nuclei were visualized by addition of anti-Guinea Pig Alexa Fluor 488 and DAPI, respectively.

### Sample Preparation for Transmission Electron Microscopy

Primary hippocampal neurons were grown on 3 mm carbon-coated sapphire disks until DIV 25, then fixed using 2% PFA with 2% glutaraldehyde in 0.1 M cacodylate buffer. Cells were postfixed with 1% osmium tetroxide, followed by 1% uranyl acetate. The samples were then dehydrated using an ethanol gradient, prior to infiltration with EmBed 812 epoxy resin (Electron Microscopy Sciences) using BDMA as an accelerator. Resin-filled BEEM capsules were attached to the cell surface to enable separation of the cell layer from the disk, and the resin was cured at 60°C for 36 h. Ultrathin sections (90 nm) were then cut by diamond knife using a Leica UC6 Ultramicrotome, and mounted on formvar-coated copper slot grids for viewing the ultrastructure.

### Acute Hippocampal Slice Preparation

Rats were deeply anaesthetized with ketamine (100 mg/kg, i.p.), the brains removed and chilled in ice-cold and oxygenated modified aCSF for which sucrose was substituted for NaCl (composition in mM: sucrose 210, glucose 20, KCl 2.5, NaH2PO4 1.25, NaHCO3 26, CaCl2 0.5, MgCl2 3, pH 7.4 when gassed with 95% O2-5% CO2). Hippocampi were dissected and slices (400 μm) cut in a manner similar to that described previously (Mockett et al., 2011) using a vibroslicer (Leica, VT1000). Slices were bathed in standard aCSF (in mM: NaCl 124, KCl 3.2, NaH2PO4 1.25, NaHCO3 26, CaCl2 2.5, MgCl2 1.3, D-glucose 10, equilibrated with carbogen 95% O2-5% CO2; 1 ml/2 h/32°C) in 24-well tissue culture dishes (one well per treatment). sAPPα and other drug treatments were subsequently applied in warmed aCSF for 15 min or 2 h. When studying inhibitor effects on sAPPα treatment, slices were pre-incubated for 30 min with the inhibitors before subsequent co-incubation with sAPPα for 2 h. When studying the effects of sAPPα treatment on pCREB expression, slices were co-incubated with inhibitors of serine/threonine-protein phosphatases (NaF, 1 mM; PMSF, 100 μM; okadaic acid, 1 μM). Following treatment, slices were washed in PBS-MC (pH 7.4) and subsequently fixed in 4% PFA in PBS-MC (pH 7.4) overnight at 4°C. Following fixation, slices were washed in PBS (pH 7.4) and embedded in 3% agarose (Roche). Slices were resliced to 50 μm sections using a vibroslicer (Vibratome 1500, Warner instruments) and stored in PBS (pH 7.4) at 4°C until use.

### Immunohistocemistry

For immunohistochemical analysis of protein in acute hippocampal slices, slices were permeabilized with 0.5% Triton X-100 in PBS (pH 7.4; 10 min). Slices were then blocked in 4% normal goat serum in PBS (pH 7.4) for 1 h at RT. Slices were incubated with primary antibodies of interest (overnight, 4°C), washed (3 × 10 min; PBS, pH 7.4) and incubated in appropriate secondary antibodies (1 h, RT), followed by 3 × 10 min washes (PBS, pH 7.4). All steps were performed with gentle agitation. Slices were mounted on coverslips (Histobond) in AquaPolymount mounting media (Polysciences) for imaging.

### Microscopy

FUNCAT-PLA images were captured with a LSM780 confocal microscope (Zeiss), using a 40x/1.4-N.A oil objective (Plan Apochromat DIC M27), a pinhole setting of 90 μm and all lasers at 2% power. The images were acquired as 8-bit mode z stacks, with the 1,024 × 1,024 pixel xy resolution through the thickness of the cell. The optical slice thickness was set to two-times oversampling, and pixel dwell times were set to 0.39–0.80 μs. For experiments examining the expression of protein using immunofluorescence, images were acquired using an Olympus IX71 inverted light microscope using a 20x/0.45-N.A objective (LUCPFLN) or 4x/0.13-N.A objective (UPFLN). The images were captured using a Hamamatsu Orca-AG camera (C4742-80-12AG) in 1024 × 1024 pixel 8-bit mode. Images were saved as.*tif* files. Electron micrographs were captured using a Philips CM100 BioTWIN transmission electron microscope with a LaB6 emitter (Philips/FEI Corporation), and images were captured using a MegaView III digital camera (Olympus).

### Image Analysis

To quantify the FUNCAT-PLA signal a custom-made ImageJ script created by Maximilian Heumüller (Max Planck Institute for Brain Research, Frankfurt) was used (tom Dieck et al., 2015), and punctate PLA signal was subsequently dilated using ImageJ for ease of viewing. To quantify immunofluorescence, neurons were outlined using ImageJ. An ‘integrated intensity/neuronal area’ value was generated for each cell and somatic compartment, including all dendrites up until intersection with neighboring dendrites. This value was corrected for average background fluorescence by subtracting average background fluorescence. Dendritic fluorescence was determined by subtracting corrected somatic values from whole cell values. Primary and secondary dendrites were analyzed by sampling gray values at 0.5 μm increments, and binning the average of each 50- and 25 μm segments, respectively. Statistical analysis was achieved by averaging each dendritic segment per cell, for a total cell average. For experiments examining pCREB expression in acute hippocampal slices, DAPI was used to define a ‘mask’ around the nuclear layer and pCREB was measured as ‘integrated intensity/DAPI area.’ For experiments examining Arc expression in acute hippocampal slices, area CA1 was defined by a square area adjacent to the hippocampal fissure (encompassing both the pyramidal cell layer and stratum radiatum) and an integrated intensity/area’ value was generated for each slice.

### Statistical Analysis

#### RT-qPCR

As the data exhibited a normal distribution (Shapiro–Wilk normality test), significance was assessed using Students *t*-tests where *P* < 0.05 was accepted as significantly different. Data were normalized to the control gene HPRT and expressed relative to no-drug control.

#### Imunocytochemistry

Statistics for all immunocytochemistry experiments were performed using Kruskal–Wallis one-way ANOVA with Dunn’s multiple comparisons test. Data were not normally distributed (D’Agostino and Pearson omnibus normality test). Outliers within the raw data sets were detected using the Grubb’s test.

#### FUNCAT-PLA

Prior to data amalgamation, outliers were removed from each experiment using Grubb’s tests, and normality was assessed (D’Agostino and Pearson omnibus normality tests). Significance was calculated on data expressed relative to control by use of one sample *t*-tests.

#### Acute Hippocampal Slices

Data for all slice work exhibited a normal distribution (Shapiro–Wilk normality test). Experiments examining pCREB expression were analyzed by use of students *t*-tests, and experiments analyzing Arc protein expression were analyzed using one-way ANOVA followed by Šidák’s multiple comparisons test. Data are expressed as fold change relative to control values.

## Discussion

Here, we show that sAPPα promotes the expression of the key plasticity protein Arc in primary neuronal cultures. Notably, Arc expression was dependent on CaMKII, MAPK, and PKG activity, which is consistent with previous reports of these kinases being essential for sAPPα’s facilitation of local protein synthesis in hippocampal synaptoneurosomes and glutamate receptor trafficking in acute hippocampal slices (Claasen et al., 2009; Mockett et al., 2019). Additionally, we have identified a critical interaction between NMDA and α7nAch receptors in triggering this effect.

Our research provides evidence of a coordinated set of signaling mechanisms through which sAPPα regulates the expression of Arc, and by association, synaptic plasticity. Similar to the well-studied neurotrophin brain-derived neurotrophic factor (BDNF), nanomolar amounts of sAPPα promotes a gradual increase in *Arc* and likewise an increase in *Zif268* mRNA. However, the relative change induced by sAPPα appears to be more modest than that of BDNF (El-Sayed et al., 2011), suggesting a neuromodulatory role for sAPPα. Further, we described a concentration-dependent effect in response to sAPPα, but not sAPPβ. While previous reports indicate that sAPPα is able to rescue morphological and plasticity-related deficits observed in APP and APP-like protein 2 (APPL2) knockout mice (Ring et al., 2007; Fol et al., 2016) and conditional APP/APPL2 NexCre knockdown mice (Li et al., 2010; Hick et al., 2015), sAPPβ is unable to ameliorate these deficits. This difference may underlie a divergence in signaling cascades between the two sAPP metabolites, further elucidating their distinct biological functions.

While previous work has described a multitude of signaling pathways mediating sAPPα-induced neuroprotection, and plasticity, of particular interest is sAPPα’s ability to increase cGMP and MAPK activity (Furukawa et al., 1996a; Gakhar-Koppole et al., 2008). Strikingly, in previous studies using isolated synapses, we have shown that sAPPα enhances *de novo* protein synthesis in a manner partially dependent on CaMKII and MAPK, and fully depending on PKG (Claasen et al., 2009). Likewise, trafficking of GluA1-containing AMPA receptors following sAPPα-enhanced LTP requires CaMKII and PKG, as well as protein synthesis (Mockett et al., 2019). Interestingly, a recently published study (Martinsson et al., 2019) has shown that knockdown or knockout of endogenous APP leads to increased levels of GluA1. Given that Arc is known to internalize GluA1- and/or GluA2-containing AMPA receptors (Chowdhury et al., 2006; Rial Verde et al., 2006), this suggests that endogenous APP or sAPPα may contribute to regulation of Arc levels (and therefore surface expression of GluA1). Building on this, and as primary hippocampal neurons express GluA2-lacking AMPA receptors (Jaafari et al., 2012), we propose that the signaling mechanisms harnessed by sAPPα not only promote increased AMPA receptor cell surface expression, but also initiate a concomitant homeostatic response whereby Arc promotes delayed AMPA receptor internalization. Future experiments should aim to explore whether this occurs globally, or specifically at activated synapses.

In the present experiments, inhibition of CaMKII significantly impaired both somatic and dendritic Arc expression, while inhibition of MAPK and PKG significantly reduced Arc expression in the dendrites alone. Thus CaMKII, MAPK, and PKG may mediate distinct aspects of sAPPα-induced functions in each neuronal compartment. Indeed, evidence suggests that PKG may act to facilitate trafficking of Rab11-positive vesicles. Rab11 is a protein primarily associated with recycling endosomes, and can mediate anterograde trafficking from the *trans*-Golgi network and perinuclear endosome (Chen et al., 1998; Ang et al., 2004; Lock and Stow, 2005; Takahashi et al., 2012). This trafficking is achieved through close association with the Ca^2+^-sensitive motor protein myosin Vb (Wang et al., 2008). Interestingly, PKG has been linked to the nitric oxide-dependent simulation of anterograde trafficking of Rab11A-positive recycling endosomes (Zhai et al., 2017) and has been shown to directly bind Rab11B (Reger et al., 2014). Importantly, Arc protein and mRNA colocalize with Rab11 (Wu et al., 2011) and inhibition of Rab11 activity impairs postsynaptic expression of Arc protein in *Drosophila* motor neurons (Ashley et al., 2018). Thus, PKG may play an important role in the activity-dependent transport of Arc-containing vesicles throughout the dendrites. Once present at the synapse, PKG, in concert with MAPK and CaMKII, may then regulate synthesis of Arc protein (Browning et al., 2000; Kleppisch et al., 2003; Atkins et al., 2004; Claasen et al., 2009; Michel et al., 2011; Mockett et al., 2011). CaMKII may also contribute to the localization of Arc protein within the somata and dendrites (Husi et al., 2000; Donai et al., 2003; Okuno et al., 2012), however, our studies do not distinguish between CaMKII isoforms. Further, it has been hypothesized that there may exist separate pools of translating and non-translating *Arc* mRNA (Steward et al., 2014) such that a pool of *Arc* mRNA may be translated within the somata, while a separate, translationally repressed pool is trafficked throughout the dendrites primed and waiting to be translated locally (Link et al., 1995). A third pool may govern rapid transcription-independent translation of *Arc* mRNA already associated with polyribosomes throughout the dendrites (Bagni et al., 2000; Na et al., 2016). Therefore, our data may reflect the presence of distinct pools of *Arc* mRNA, regulated differentially by CaMKII, MAPK, and PKG. Additionally, recent work has identified a novel role for Arc protein through shared properties with retroviral Gag proteins: Arc forms virus-like capsids capable of encapsulating and transporting functional RNA and protein between cells in endosomal-derived extracellular vesicles (Pastuzyn et al., 2018), therefore suggesting Arc can modulate synapse-, cell-, and network-wide plasticity.

A crucial, yet outstanding question in the literature is the identity of the ‘sAPPα receptor.’ Previous evidence has described a role of GABA_B_ (Rice et al., 2017, 2019), Na/K ATP’ase (Dorard et al., 2018), α7nAch (Richter et al., 2018) and NMDA receptors (Gakhar-Koppole et al., 2008; Mockett et al., 2019) in mediating sAPPα’s plasticity enhancing effects. To understand the receptors contributing to the facilitated Arc expression, we investigated the possible contributions of these and other candidate receptors (mGluRI/II and TrkB) due to their similar neuromodulatory or plasticity-promoting properties (Raymond et al., 2000; Mockett et al., 2011). Our data suggest a synergistic effect between activation of NMDARs and α7nAChRs. Sole inhibition of either receptor alone led to a partial impairment in the sAPPα-mediated expression of Arc protein, whereas simultaneous inhibition of both receptors fully eliminated the enhancement of Arc protein expression in the dendrites. Further, we have extended our studies to the more complex biological system of acute hippocampal slices. Here, our findings closely reflected that of the primary hippocampal culture work, as sAPPα significantly enhanced Arc protein expression in area CA1 of the hippocampus in a manner dependent on activation of NMDA and α7nACh receptors. This finding was corroborated by an increase in pCREB, a downstream NMDAR signaling event (Xia et al., 1996), in area CA1. Importantly, many of the reports describing sAPPα’s ability to enhance LTP has been shown in CA1, including the dependence on NMDAR (Mockett et al., 2019) and α7nACh receptors (Richter et al., 2018). The mechanism governing this effect may involve protein–protein interactions between α7nAChR-NMDAR complexes (Li et al., 2012, 2013), or direct binding of sAPPα to NMDA or α7nAChR (Cousins et al., 2009; Innocent et al., 2012; Forest et al., 2018). Likewise, trafficking of NMDA receptors has been observed in response to sAPPα (Mockett et al., 2019) and may occur through α7nAChR activation (Marchi et al., 2015). This therefore may be a mechanism which provides additive Ca^2+^ or activation of downstream signaling pathways (Aramakis and Metherate, 1998; Brunzell et al., 2003; Gould and Lewis, 2005). A similar synergistic effect has been found between the activation of NMDARs and Gs-coupled dopamine and β-adrenergic receptors in the PKA-dependent increase in Arc protein (Bloomer et al., 2008). PKG-dependent NMDAR activation has been shown to enhance the expression of IEG Arc, c-Fos and Zif268 in the lateral amygdala *in vivo* in response to both fear conditioning (Ota et al., 2010) and LTP-inducing stimulation (Ping and Schafe, 2010) and inhibition of neuronal nitric oxide (nNOS), soluble guanylyl cyclase (sGC), or PKG attenuated the bicuculline-induced expression of Arc, c-Fos and Zif268 in neuronal cultures (Gallo and Iadecola, 2011). Further, both NMDAR and α7nAch can facilitate CaMKII and MAPK activation (Cammarota et al., 2000; Thalhammer et al., 2006; Gubbins et al., 2010). Therefore, concerted activity between NMDARs and α7nAChRs may act to promote sAPPα-mediated Arc expression through synergistic activation of downstream cascades involving CaMKII, PKG and MAPK.

sAPPα shows hallmark neuroprotective and neurotrophic properties and is capable of stimulating neurite outgrowth, regulating spine morphology (Clarris et al., 1994; Hick et al., 2015), enhancing synaptodendritic protein synthesis, and facilitating protein synthesis-dependent LTP (Taylor et al., 2008; Claasen et al., 2009; Moreno et al., 2015; Mockett et al., 2019), as well as protecting against excitotoxic injury *in vitro* and *in vivo* (Mucke et al., 1996; Ryan et al., 2013). Likewise, the synthesis and function of Arc protein has been previously linked to many of these characteristics, such as stimulation of CaMKII-dependent neurite extension (Donai et al., 2003), the homeostatic intergration of synapse downscaling following seizure activity and chronic excitation (Lyford et al., 1995; Shepherd et al., 2006; Gao et al., 2010) with increased morphological plasticity and spine size (Messaoudi et al., 2007; Peebles et al., 2010), and its role in facilitating LTP (Guzowski et al., 2000; Plath et al., 2006; Messaoudi et al., 2007; Nakayama et al., 2016). Our studies provide insight into how sAPPα promotes the expression of these mechanisms through the utilization of Arc protein. It is important to note that we have titrated sAPPa concentrations and found that 1 nM is sufficient to initiate a variety of molecular events (Claasen et al., 2009; Ryan et al., 2013; Mockett et al., 2019). There is, however, only limited knowledge of the physiological levels of sAPPα and to what extent they are dynamically regulated *in vivo*.

## Conclusion

This work extends the current understanding of possible mechanisms through which sAPPα enhances synaptic plasticity. Here we have described an enhancement of Arc protein synthesis driven by activation of NMDA and α7nAch receptors, and downstream activation of CaMKII, MAPK, and PKG. sAPPα levels are reduced in many disease states and neurological disorders, many of which involve impaired or altered plasticity (Lannfelt et al., 1995; Kim et al., 2009; Selnes et al., 2010; Jakobsson et al., 2013; Miyajima et al., 2013; Rolstad et al., 2015). Therefore, furthering our understanding of the underlying processes governing sAPPα’s regulation of key plasticity proteins, including Arc, is a fundamental step in understanding dysfunction which arises in many cognitive diseases.

## Data Availability

The raw data supporting the conclusions of this manuscript will be made available by the authors, without undue reservation, to any qualified researcher.

## Author Contributions

RL was the major contributor to the experimental aspects of the study, contributed to the design of the study, prepared the primary neuronal cultures, isolated RNA, carried out the RT-qPCR and immunochemistry experiments, analyzed and interpreted the corresponding data, and drafted the manuscript. ME carried out the FUNCAT-PLA study and the corresponding data analysis and critically revised the manuscript. MB and CW carried out immunochemistry experiments, analyzed and interpreted the corresponding data. KP produced recombinant sAPPα, analyzed and interpreted the corresponding data. WT designed the protocols for recombinant sAPPα production, analyzed and interpreted the corresponding data, critically reviewed the manuscript. WA critically revised the manuscript. JW conceived and participated in the design and co-ordination of the study, undertook data analysis and interpretation, and critically assessed the manuscript.

## Conflict of Interest Statement

The authors declare that the research was conducted in the absence of any commercial or financial relationships that could be construed as a potential conflict of interest.
