# Supplementary material for: Secreted Amyloid Precursor Protein-Alpha Promotes Arc Protein Synthesis in Hippocampal Neurons
Source: Front Mol Neurosci. 2019 Aug 14;12:198. doi: 10.3389/fnmol.2019.00198 (PMC6702288; doi:10.3389/fnmol.2019.00198)
Supplement: Supplementary file 1 [file Data_Sheet_1.docx]

SECRETED AMYLOID PRECURSOR PROTEIN-ALPHA PROMOTES ARC PROTEIN SYNTHESIS IN HIPPOCAMPAL NEURONS

Rhys W. Livingstone^1^, Megan K. Elder^1^, Maya C. Barrett^1^, Courteney M. Westlake^1^, Katie Peppercorn^3^, Warren P. Tate^3^, Wickliffe C. Abraham^2^, Joanna M. Williams^1*^

^1^Department of Anatomy, ^2^Department of Psychology, ^3^Department of Biochemistry,

Brain Health Research Centre, Brain Research New Zealand, Rangahau Roro, University of Otago, Dunedin, New Zealand

*** Correspondence:**Assoc. Prof. Joanna Williams
joanna.williams@otago.ac.nz

Supplementary Material

## Supplementary Figures


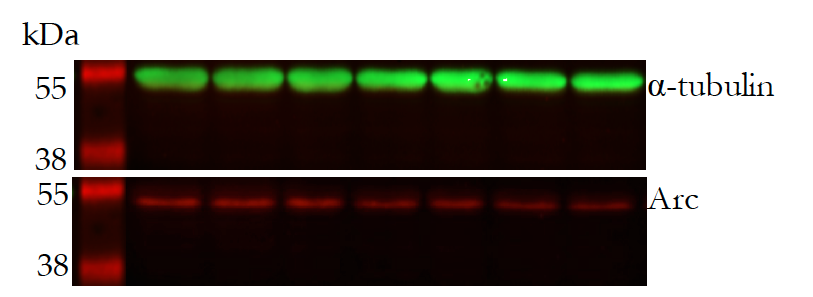


**Supplementary Figure 1.** **Specificity of Arc Primary Antibody.** Western blot analysis of protein extracted from primary hippocampal cultures grown in 6-well plates (25 µg per lane). Upper panel shows anti-α-tubulin immunoreactivity, visualized with IRDye800. Lower panel shows anti-Arc immunoreactivity visualized with IRDye 680. Anti-Arc antibody generates a single band at approximately 50 kDa. Left lane: 55 and 38 kDa molecular weight markers (Rainbow Marker; GE Healthcare Life Sciences) are visible.


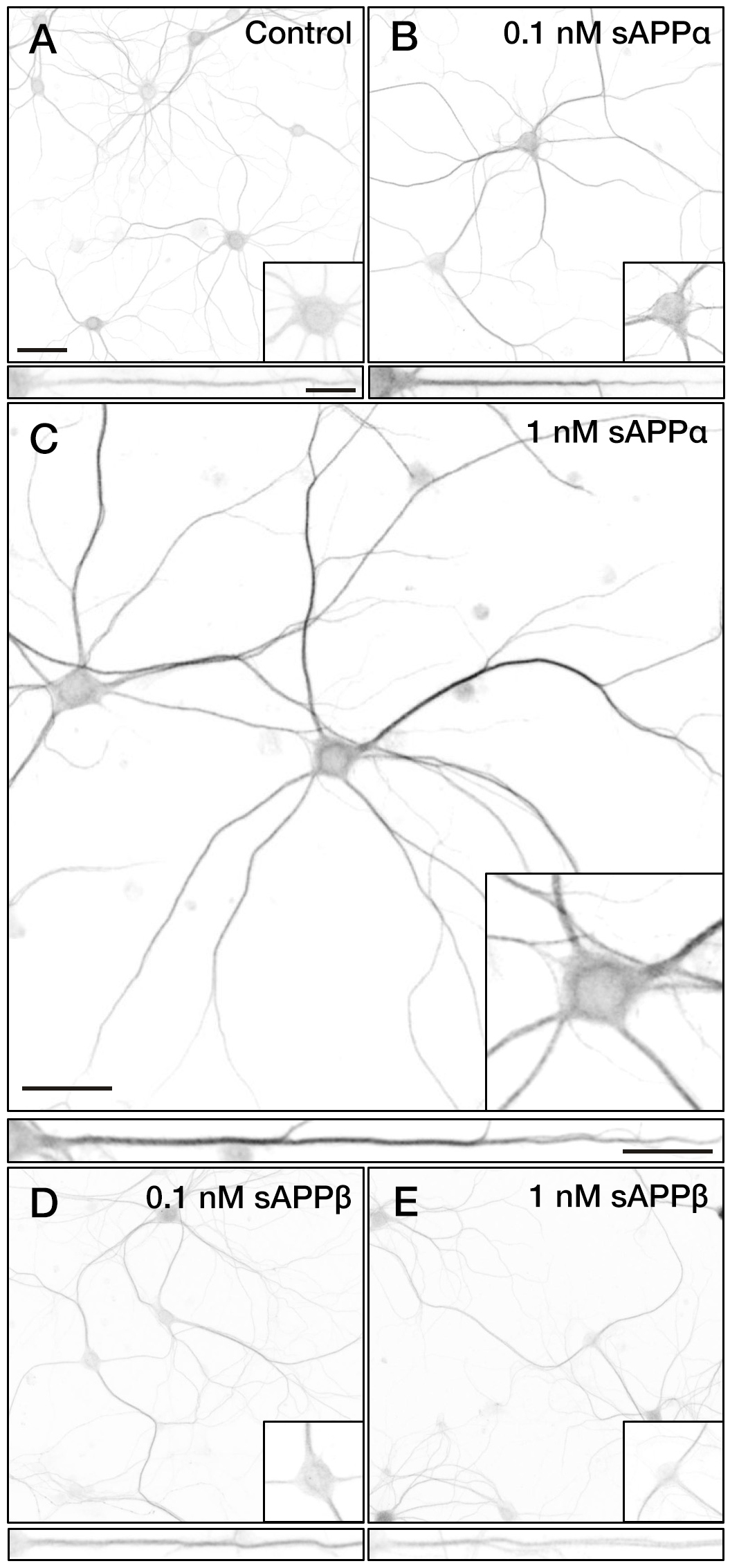


**Supplementary Figure 2.** **Light micrographs of Arc immunoreactivity following sAPPα and sAPPβ treatment.** Representative fluorescence images showing primary hippocampal neurons in A) basal condition, or following B) 0.1 nM sAPPα, C) 1 nM sAPPα, D) 0.1 nM sAPPβ, or E) 1 nM sAPPβ. Arc immunoreactivity is detected using a goat anti rabbit Alexa Fluor 555 and is expressed as greyscale for ease of visualisation. Scale bar = 50 μm. Inset images show magnified somatic compartments and lower panels show magnified dendritic compartments (scale bar = 100 μm).

**
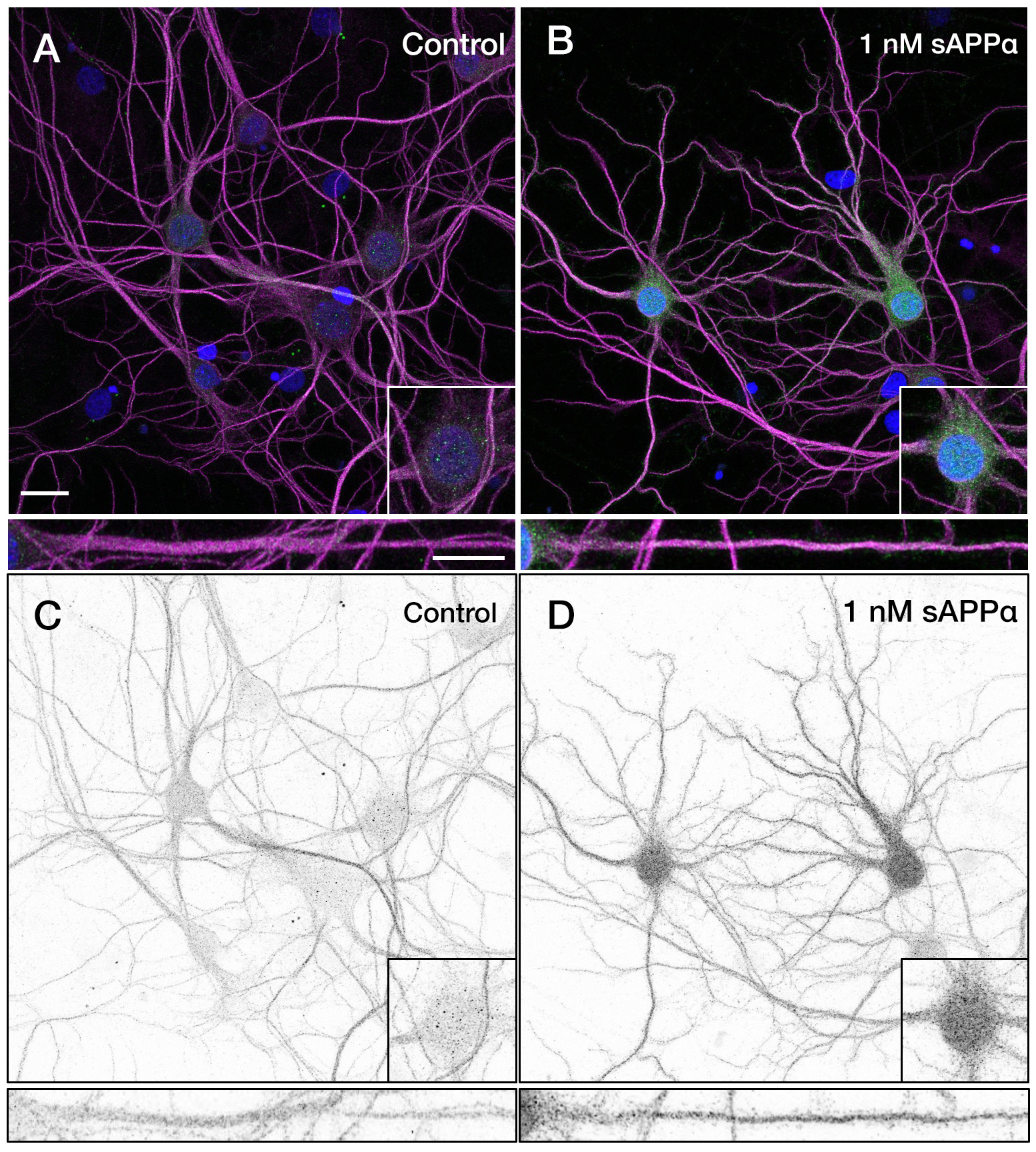
**

**Figure 3.** **Confocal** **micrographs of Arc immunoreactivity following sAPPα treatment.** Representative confocal images showing primary hippocampal neurons in A) basal condition, or following B) 1 nM sAPPα. Arc immunoreactivity is detected using a goat anti rabbit Alexa Fluor 555 and is expressed as green with MAP2 (magenta) and DAPI blue). Grayscale images show Arc expression during C) basal state and following D) 1 nM sAPPα, for ease of visualisation. Scale bar = 50 μm. Inset images show magnified somatic compartments and lower panels show magnified dendritic compartments (scale bar = 10 μm).

## Supplementary Methods

*Protein Extraction*

Primary hippocampal cultures were grown for protein harvesting on Poly-D-lysine coated wells of a 6-well plate, and harvested at DIV24-27. Following treatment, the cell layer was washed (PBS-MC, pH 7.4), scraped from the well and collected by centrifugation (14,000 x *g*, 5 min, 4 °C). The pellet was washed with PBS and centrifuged again before the cells were lysed and homogenised in buffer (1 mM EGTA, 1 mM EDTA, 0.1 mM PMSF, 1 x Complete, 1 % Triton-X, 10 % SDS, 10 μM KN-62 in PBS). The samples were sonicated (Bandelin Sonorex Digital Ultrasonic bath; 2 min, RT), and centrifuged (20 min, 14 000 x *g*, 4 °C). The supernatant was collected, and the pellet was re-homogenised. Protein was quantified using the BCA assay and stored at - 80 °C until use.

*Western Blot analysis*

SDS-Polyacrylamide resolving gels (9% Bis-Acrylamide (Bio-Rad), 0.5 mM SDS, 0.05% TEMED, 0.05% APS buffered with 0.37 M Tris-HCl) were cast in NOVEX 1 mm gel cassettes (ThermoFisher). The gels were sealed with water overnight at 4 °C. Stacking gels (5% acrylamide, 0.5 mM SDS, 0.1% TEMED, 0.05% APS buffered with 0.125 M Tris-HCl) were cast, and the gels were left at RT for 20 min. NOVEX cassettes were locked into a NOVEX X-cell Surelock Minicell system (Invitrogen). Inner and outer chambers of the box were filled with sufficient running buffer (192 mM glycine, 0.1% SDS buffered with 25 mM Tris-HCl) to cover the wells in the gels. The prepared samples were pipetted into the wells alongside a molecular weight rainbow marker (Amersham, GE Healthcare Life Sciences). Electrophoresis was conducted at 125 V for 2 h, or until the dye front reached the base of the gel. The separated proteins were then transferred to nitrocellulose membranes (Amersham Protran 0.45 μm NC, GE Healthcare Life Science) using NOVEX X-cell II blotting modules (Invitrogen). The transfer was carried out at 100 mAmp for 2 h, using transfer buffer (96 mM glycine, 10 % methanol buffered with 12 mM Tris-HCl).

Following transfer, blocking buffer was applied to the membranes (Odyssey blocking buffer; Li-Cor; 1 h, RT). Primary antibodies were diluted in 0.1 % BSA, 0.1 % NGS in PBS with 0.1% Tween, and membranes were probed overnight at 4 °C with gentle rocking. The following day, unbound antibody was removed using PBS-Tween (4 x 5 min) and the membranes were incubated with the appropriate secondary antibodies in PBS-Tween (1 h, RT, gentle rocking). Unbound antibody was washed using PBS-Tween (4 x 5 min) and the membranes were washed in PBS, before being allowed to dry on filter paper prior to scanning.

Protein was detected using the LI-COR Odyssey Infrared Fluorescence Imaging system. Image capture was carried out using Image Studio. The brightness and contrast levels of captured images were adjusted for image presentation.
